# Social and emotional developmental vulnerability at age five in Aboriginal and non-Aboriginal children in New South Wales: a population data linkage study

**DOI:** 10.1186/s12939-019-1019-x

**Published:** 2019-07-31

**Authors:** Anna Williamson, Alison Gibberd, Mark J. Hanly, Emily Banks, Sandra Eades, Kathleen Clapham, Kathleen Falster

**Affiliations:** 1 Melbourne School of Population and Global Health, Centre for Epidemiology and Biostatistics, The University of Melbourne, Melbourne, Australia; 2 Centre for Big Data Research in Health, University of New South Wales, Kensington, Australia; 3 National Centre for Epidemiology and Population Health, Australian National University, Canberra, Australia; 4 The Sax Institute, PO Box K617, Haymarket, NSW 1240 Australia; 5 Australian Health Services Research Institute (AHSRI), University of Wollongong, Wollongong, Australia; 6 Centre for Social Research Methods, Australian National University, Canberra, Australia

**Keywords:** Mental health, Early childhood development, Indigenous population, Linked administrative data

## Abstract

**Background:**

Early childhood social and emotional development underpins later social, emotional, academic and other outcomes. The first aim of this study was to explore the association between child, family and area-level characteristics associated with developmental vulnerability, amongst Aboriginal and non-Aboriginal children in their first year of school. The second aim was to quantify the magnitude of the social and emotional developmental inequalities between Aboriginal and non-Aboriginal children and the extent to which differences in socioeconomic disadvantage and perinatal characteristics explained this inequality.

**Methods:**

This retrospective cohort study used cross-sectoral data linkage to identify and follow participants from birth to school age. In this way, social and emotional development was examined in 7,384 Aboriginal and 95,104 non-Aboriginal children who were included in the Australian Early Development Census in their first year of full-time school in New South Wales (NSW) in 2009 or 2012 and had a birth registration and/or perinatal record in NSW. The primary outcome measures were teacher-reported social competence and emotional maturity as measured using the Australian version of the Early Development Instrument.

**Results:**

The mean age at the start of the school year for children in the study sample was 5.2 years (SD = 0.36 years). While 84% of Aboriginal children scored favourably - above the vulnerability threshold – for social competence and 88% for emotional maturity, Aboriginal children were twice as likely as non-Aboriginal children to be vulnerable on measures of social development (RR = 2.00; 95%CI, 1.89–2.12) and had 89% more risk of emotional vulnerability (RR = 1.89; 95%CI, 1.77–2.02). The inequality between Aboriginal and non-Aboriginal children was largely explained by differences in the socioeconomic and perinatal health characteristics of children and families. Thus, after adjusting for differences in measures of socioeconomic advantage and disadvantage (Model 2), the relative risk was attenuated to 1.31 (95% CI: 1.23–1.40) on the social competence domain and 1.24 (95% CI, 1.15–1.33) on the emotional maturity domain. Child, family and area-level characteristics associated with vulnerability were identified.

**Conclusions:**

Most of the gap in early childhood social and emotional development between Aboriginal and non-Aboriginal children can be attributed to socioeconomic and early life health disadvantage. Culturally safe health and social policies addressing the socioeconomic and health inequalities experienced by Aboriginal children are urgently required.

**Electronic supplementary material:**

The online version of this article (10.1186/s12939-019-1019-x) contains supplementary material, which is available to authorized users.

## Strengths and limitations of this study

- To our knowledge, the current study is the largest and most comprehensive of social and emotional development among Australian Aboriginal children to date.
- By linking several cross-sectoral population level data sources, we were able to examine a broader range of child, family and area level characteristics – compared with information available from a single data source – that were associated with social and emotional development in Aboriginal and non-Aboriginal children, which is useful for targeting early intervention services.
- The use of multiple linked datasets, as in the current study, increases enumeration of Aboriginal people, compared with single data sources.
- While the measures of socioeconomic advantage and disadvantage used in this study provided powerful insights into the factors underpinning emotional and social development amongst Aboriginal children, they were limited to information available in routinely collected data.

## Introduction

Social and emotional development in early childhood has been defined as the evolving ability to “form close and secure adult and peer relationships; experience, regulate, and express emotions in socially and culturally appropriate ways; and explore the environment and learn — all in the context of family, community, and culture” (Yates et al., 2008, p. 2). It is increasingly recognised that social and emotional development in early childhood plays an important role in the successful transition to school and is also related to later academic achievement, mental health and wellbeing [1]. Most Aboriginal children have good mental health [2]. However, the social and health disadvantage [3, 4] that Aboriginal Australians experience from the perinatal period is well documented. Little is known about the early life characteristics of Aboriginal children and their families that are related to social and emotional development. This evidence is urgently needed to identify opportunities to promote social and emotional development in early childhood [5], when supportive interventions have been shown to be particularly effective [6, 7].

In recognition of the acute and long-term importance of early childhood development, the Australian Government has implemented a triannual census of children entering the first year of school since 2009 [8]. Data for the census are collected via teacher-completion of the Australian Early Childhood Development Census (AEDC) (formerly the Australian Early Development Index) for each student. The AEDC covers five developmental domains, two of which are of particular relevance to social and emotional wellbeing: social competence (exemplified by the ability to get along with and respect peers and teachers, exert self-control, demonstrate curiosity and work independently) and emotional maturity (exemplified by recognising and responding to others’ moods appropriately and inviting other children to play). Data emerging from the AEDC has revealed inequalities in social and emotional development between Aboriginal and non-Aboriginal children throughout Australia [9, 10]. While two studies have used the AEDC to examine early childhood social and emotional development in the general population of children [10, 11], to our knowledge, there have been no studies focused specifically on Aboriginal children and the early life child, family and community characteristics associated with their increased risk of social and emotional vulnerability.

In this paper, we extend on previous work in this field by drawing on the Seeding Success data resource [12] which allows a detailed examination of the factors associated with social and emotional development amongst Aboriginal school entrants in New South Wales, Australia. The resource includes linkage of 2009 and 2012 AEDC data with routinely collected data from perinatal, birth and Public School Enrolment records. First, we explored the association of a range of child, family and residential area-level characteristics (such as socio-economic indices for areas and level of remoteness) associated with developmental vulnerability, with a view to gaining insights to improve outcomes for Aboriginal children, including who might benefit most from enhanced support to improve social and emotional development. Second, we quantified the magnitude of the social and emotional developmental inequalities between Aboriginal and non-Aboriginal children and examined the extent to which differences in socioeconomic disadvantage and perinatal characteristics explained this inequality.

## Methods

This study was a retrospective cohort study using cross-sectoral data linkage.

### Data sources, data linkage and linked data resource

The 2009 and 2012 AEDC included 97% of children enrolled in their first year of school in the state of New South Wales (NSW), Australia’s most populous state. As part of the broader ‘Seeding Success’ study, the NSW Centre for Health Record Linkage probabilistically linked the AEDC data to a number of other NSW population-based datasets, including perinatal records (the Perinatal Data Collection), birth registrations from the Register of Births, Deaths and Marriages, hospital admissions (the Admitted Patient Data Collection) and Public School Enrolment records. More detailed information about these data sources, data linkage, and the cohort of children in the Seeding Success study have been published previously elsewhere [12, 13]. Briefly, the Seeding Success data resource comprises data for all children with a linked NSW perinatal record and/or birth registration, who were in their first year of school and had an AEDC record in 2009 or 2012 or were in a top-up sample in 2010 which the AEDC undertook to ensure sufficient number to publish area-level data for areas with small population size (*n* = 166,278 children).

### Study population for analysis

**Fig. 1:**
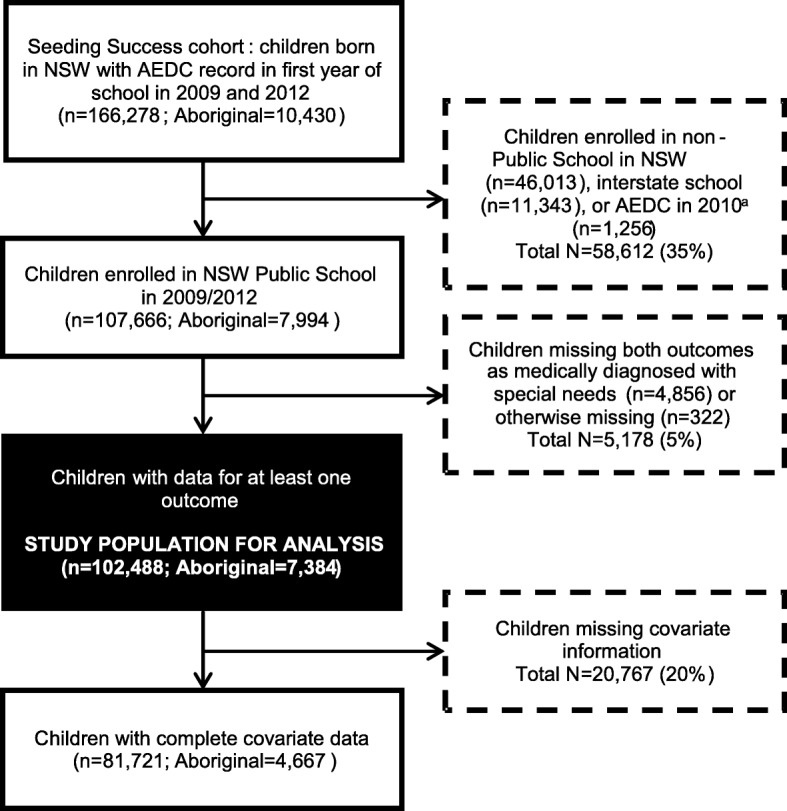
Inclusion and exclusion criteria for selection of the study population

From the 166,278 children in the Seeding Success data resource, we restricted our analysis to children who started school in a NSW Public School in 2009 or 2010, because information about parent/carer education and occupation was collected and available for these children. To this end we excluded children enrolled in non-Public Schools in NSW (*n* = 46,013 children), interstate schools (*n* = 11,343 children) or who were in the 2010 AEDC top-up sample (n = 1,256 children). (Fig. 1). We then also excluded children who were missing data for both outcomes, either because they had medically diagnosed special needs (n = 4,856 children) or they were missing this data for unknown reasons (*n* = 322 children). The binary outcomes used in this study are not available for children with special needs as the AEDC has not been validated in this population. Of the remaining 102,488 children in the study population, 7,384 (7%) were Aboriginal.

### Social and emotional development outcomes

The AEDC is a population-level measure of early childhood development on five domains, as assessed by a teacher within 4–6 months of the child commencing school, gathered using the Australian version of the Early Development Instrument (AvEDI). Both instruments have been found to be valid and reliable [14–19]. The AvEDI has also been validated among indigenous Australian children [20]. For this study, we used the social competence and emotional maturity domains as the study outcomes. AvEDI domain scores are positively skewed, so we used binary outcomes (i.e. developmentally vulnerable or not) for each domain. Following the national standard, children with domain scores below the first decile cut-point for the 2009 AvEDI domain scores were categorised as developmentally vulnerable [8].

### Exposure variables

We categorised children as Aboriginal and/or Torres Strait Islander (hereafter referred to as Aboriginal in accordance with accepted terminology in New South Wales [21]) if they, or either parent, were recorded as such on the child’s birth registration, perinatal or hospital birth records, or the AEDC [12]. Other variables included in this analysis were child’s sex, whether the mother was married/partnered at childbirth, private health patient/insurance status at childbirth, maternal school education, the highest level of parental occupation(s), attendance at preschool/childcare in the year prior to school, AEDC census year, maternal age at childbirth, parity, smoking during pregnancy, antenatal care in the first 20 weeks of pregnancy, maternal comorbidity during pregnancy (including pre-existing and gestational hypertension and/or diabetes), and child’s gestational age. Geographical remoteness, measured by the Accessibility/Remoteness Index of Australia (ARIA+), [22] and quintiles of socio-economic disadvantage, measured by the Australian Bureau of Statistics’ Index of Relative Socio-economic Advantage and Disadvantage, [23] were assigned based on the mother’s place of residence at the child’s birth.

### Missing data

The majority of variables contained valid data for over 95% of the study population (Additional file 1: Table S1). Of the 102,488 children in the study population, 20,767 (20%) children were missing data for one or more of the analysis variables. Among the 7,384 Aboriginal children, 2,717 (37%) children were missing information. Missing data were more common for information collected at school enrolment: maternal school education (19% for Aboriginal and 8% for non-Aboriginal children); parental occupation (16 and 6%); and preschool/childcare attendance (9 and 6%). To optimise the use of available data and minimise bias associated with inclusion of children with complete data only, we assumed the data were missing at random and undertook multiple imputation via chained equations [24], generating five complete datasets using Stata 12.1 [25].

### Statistical analysis

We first estimated the proportion of Aboriginal and non-Aboriginal children who were vulnerable on each domain. To examine associations between each outcome and child, family and area-level characteristics in Aboriginal children and non-Aboriginal children, we fitted Poisson regression models with a robust error variance using PROC GENMOD in SAS 9.4 [26] separately for both Aboriginal and non-Aboriginal children, adjusted for sex and AEDC year.

To examine the magnitude of the absolute and relative inequalities in social and emotional developmental vulnerability between Aboriginal and non-Aboriginal children, we estimated the relative risks and risk differences for both outcomes for Aboriginal compared to non-Aboriginal children in the study population. We fitted Poisson regression models with a robust error variance using PROC GENMOD in SAS 9.4, with a log link to obtain the relative risks, and an identity link for the risk differences (baseline model only) [26]. Our baseline model adjusted for sex and AEDC census year (Model 1). We did not adjust for the child’s age as the AvEDI domain outcomes already take age into account. To investigate whether child, family and area-level indicators of socioeconomic advantage and disadvantage explained any of the observed inequalities between Aboriginal and non-Aboriginal children, we further adjusted for private health insurance/public patient, mother married or partnered at the child’s birth (yes/no), maternal school education (12 years, 10–11 years or ≤ 9 years completed), highest occupation level of either parent (managers/professionals, business managers/associated professionals, trades/clerks/services, drivers/hospitality/labourers, and not in paid work in the last 12 months), area-level socioeconomic disadvantage (quintiles from least disadvantaged to most disadvantaged), and geographic remoteness (major city, inner regional, outer regional, and remote/very remote) in Model 2. To investigate whether differences in perinatal factors further explained the inequalities, we added maternal age at childbirth (< 20, 20–24, 25–29, 30–34, and ≥ 35 years), parity (no prior births, one prior birth, and two or more prior births), smoking during pregnancy (yes/no), antenatal care in the first 20 weeks (yes/no), maternal comorbidity during pregnancy (yes/no), and gestational age (22–33, 34–36, 37–38, and ≥ 39 weeks) to Model 3, in addition to Model 2 covariates.

All models were estimated on each of the imputed datasets and the regression estimates were then pooled using Rubin’s rules using PROC MIANALYZE in SAS 9.4. [27]

### Ethical approval

Ethical approval was obtained from the NSW Population Health Services and Research Ethics Committee (2014/04/523), the NSW Aboriginal Health and Medical Research Council Ethics Committee (1031/14) and the Australian National University Human Research Ethics Committee (2014/384).

## Results

Of the total 102,488 children, 7,384 (7%) were Aboriginal. Aboriginal children were more likely than their non-Aboriginal peers to have mothers who finished their education at Year 9 or less (19% versus 6%) and carers who were not in the paid workforce (31% versus 8%) (Table 1). The mothers of Aboriginal children were also more likely to have had 2 or more previous pregnancies (39% versus 24%) and to have been aged less than 20 years at the time of their child’s birth (17% versus 3%). Most Aboriginal and non-Aboriginal children attended preschool (81 and 89%, respectively). The majority of all mothers accessed antenatal care in the first 20 weeks of pregnancy (81 and 89%, respectively) and most children were born at full term (68 and 72%, respectively) (explored in more detail elsewhere [28]).

**Table 1:** Characteristics of Aboriginal and non-Aboriginal children in their first year of school in New South Wales in 2009 and 2012^h^

	Aboriginal^a^		Non-Aboriginal		Total	
	n	%	n	%	n	%
Total (N)	7384	100	95104	100	102488	100
**Socio-demographic characteristics**						
Female sex	3760	51	46784	49	50544	49
Mother married/partnered at child’s birth^b^	3505	47	79302	83	82807	81
Private patient/insurance at child’s birth^b^	511	7	31579	33	32090	31
Maternal school education^c^						
12 years	2140	29	61148	64	63288	62
10–11 years	3820	52	28691	30	32511	32
≤ 9 years	1424	19	5266	6	6690	7
Highest level of parental occupation^d^						
Managers/professionals	613	8	24809	26	25422	25
Business managers/associated professionals	816	11	23133	24	23949	23
Trades/clerks/services	1607	22	24060	25	25667	25
Drivers/hospitality/labourers	2049	28	15591	16	17640	17
Not in paid work in last 12 months	2299	31	7511	8	9810	10
Area-level disadvantage^e^						
Quintile 5 (Least disadvantaged)	378	5	23884	25	24262	24
Quintile 4	914	12	20211	21	21125	21
Quintile 3	3096	42	32697	34	35793	35
Quintile 2	1389	19	10072	11	11461	11
Quintile 1 (Most disadvantaged)	1607	22	8240	9	9847	10
Geographic remoteness^f^						
Major City	2815	38	62858	66	65673	64
Inner Regional	2578	35	23985	25	26563	26
Outer Regional	1554	21	7709	8	9263	9
Remote/Very Remote	437	6	552	1	989	1
Attended preschool/childcare in year before school	5974	81	84547	89	90521	88
AEDC census year						
2009	3325	45	45180	48	48505	47
2012	4059	55	49924	52	53983	53
**Perinatal characteristics**						
Maternal age at childbirth (years)						
< 20	1254	17	3190	3	4444	4
20–24	2254	31	13871	15	16125	16
25–29	1856	25	26177	28	28033	27
30–34	1344	18	31698	33	33042	32
≥ 35	675	9	20167	21	20842	20
No prior births	2475	34	39395	41	41870	41
One prior birth	2057	28	33081	35	35138	34
Two or more prior births	2852	39	22628	24	25480	25
Smoking during pregnancy	3549	48	13419	14	16968	17
Antenatal care in first 20 weeks of pregnancy	5954	81	84951	89	90905	89
Maternal comorbidity during pregnancy^g^	702	10	10383	11	11085	11
Gestational age						
Early preterm (22–33 weeks)	187	3	1397	1	1584	2
Late preterm (34–36 weeks)	533	7	4464	5	4997	5
Early term (37–38 weeks)	1669	23	20995	22	22664	22
Full to postterm (≥39 weeks)	4995	68	68248	72	73243	71

The counts are an average from the 5 complete datasets following multiple imputation of missing data. Details of the missing data are in Additional file 1 Table S1. ^a^ Defined as child or parent identified as Aboriginal on any of the birth records (i.e. perinatal data collection, birth registration or hospital birth record), or AEDC school record; ^b^ Based on hospital birth record; ^c^ Highest level of education of mother recorded on school enrolment (high school completion is equivalent to 12 years); ^d^ Based on highest ranking occupation of either parent or carer recorded on school enrolment; ^e^ Socio-Economic Indices for Areas (SEIFA) Index of Relative Socio-economic Advantage and Disadvantage population quintiles based on mother’s statistical local area of residence at the time of birth; ^f^ Accessibility/Remoteness Index of Australia (ARIA+) based on mother’s statistical local area of residence at the time of birth; ^g^ Includes pre-existing and gestational-onset diabetes and hypertension

^h^Imputed data is included where appropriate

### Social competence and emotional maturity amongst aboriginal and non-aboriginal school entrants

**Fig. 2:**
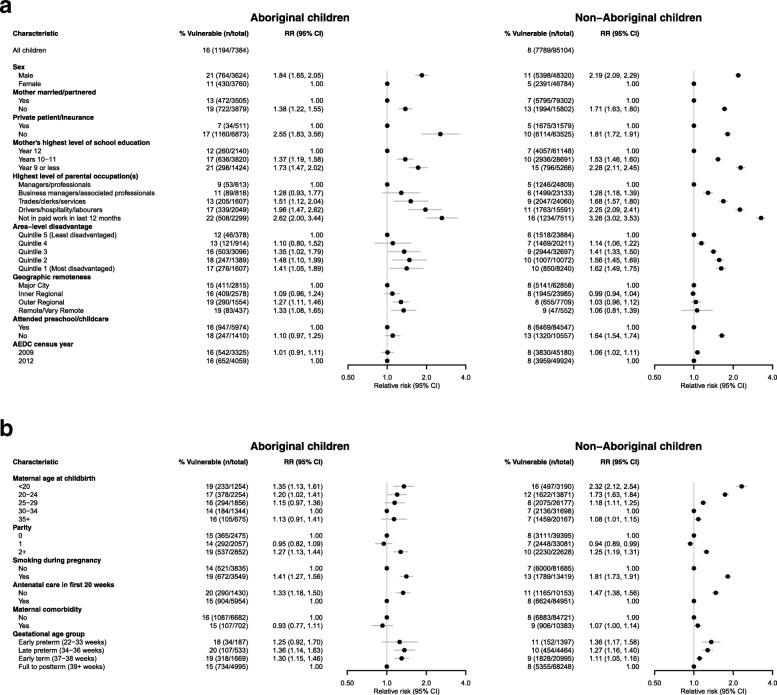
**a** Relative risks of developmental vulnerability on the social competence domain for socio-demographic and perinatal characteristics of Aboriginal children in their first year of school in New South Wales in 2009 and 2012, adjusted for sex. **b** Relative risks of developmental vulnerability on the social competence domain for socio-demographic and perinatal characteristics of non-Aboriginal children in their first year of school in New South Wales in 2009 and 2012, adjusted for sex

**Fig. 3:**
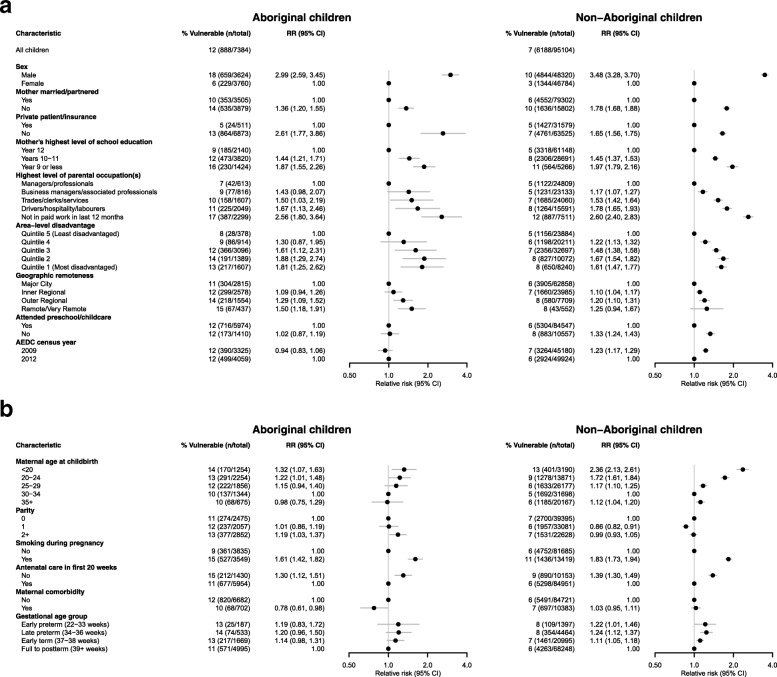
**a** Relative risks of developmental vulnerability on the emotional maturity domain for socio-demographic and perinatal characteristics of Aboriginal children in their first year of school in New South Wales in 2009 and 2012, adjusted for sex. **b** Relative risks of developmental vulnerability on the emotional maturity domain for socio-demographic and perinatal characteristics of non-Aboriginal children in their first year of school in New South Wales in 2009 and 2012, adjusted for sex

Eighty-four percent of Aboriginal children scored above the cut-point for developmental vulnerability in terms of social competence, as did 92% of their non-Aboriginal peers. Thus 16% of Aboriginal and 8% of non-Aboriginal kindergarten students were developmentally vulnerable on the social competence domain (Fig. 2a). Eighty-eight percent of Aboriginal children scored above the cut point for developmental vulnerability in relation to emotional maturity, as did 93% of their non-Aboriginal peers. Twelve percent of Aboriginal children were thus found to be vulnerable on the emotional maturity domain compared to 7% of their non-Aboriginal peers (Fig. 3a).

### Factors associated with risk of developmental vulnerability in relation to social competence

#### Aboriginal children

After adjusting for census year and sex, a range of child and family characteristics were associated with an increased risk of developmental vulnerability on the social competence domain in Aboriginal children (Fig. 2a and b). Social vulnerability was almost twice as likely among Aboriginal boys than girls (relative risk (RR) 1.84, 95% confidence interval (CI): 1.65–2.05). A range of family level factors including not having private health insurance, low maternal education and carers not being in paid work were also associated with an increased risk of social vulnerability. Perinatal factors, including younger maternal age and smoking in pregnancy were also associated with higher risk. In terms of area-level variables, children who lived in the most disadvantaged areas were at greater risk of social competence vulnerability than children in the most advantaged areas (e.g. 17% in the most disadvantaged quintile versus 12% in the most advantaged, RR: 1.41, 95% CI: 1.05–1.89), and the risk of vulnerability was higher in more remote areas.

#### Non-aboriginal children

Non-Aboriginal boys were more than twice as likely as non-Aboriginal girls to be classified as vulnerable in relation to social competence (RR: 2.19, 95% CI: 2.09–2.29). Family level factors including younger maternal age, low maternal education and carers not being in paid work were also associated with increased risk of social vulnerability amongst non-Aboriginal children. Geographic remoteness was not associated with social competence vulnerability amongst this group, however, living in a disadvantaged area was. Non-Aboriginal children who did not attend preschool were also at significantly greater risk of social competence vulnerability.

### Factors associated with risk of developmental vulnerability in relation to emotional maturity

#### Aboriginal children

Following adjustment for census year and sex, a range of child and family characteristics were significantly associated with vulnerability on the emotional maturity domain among Aboriginal children (Fig. 3a and b). Aboriginal boys were significantly more likely than Aboriginal girls to be assessed as vulnerable (18% versus 6%, RR: 2.99, 95% CI: 2.59–3.45). Family factors associated with emotional vulnerability included absence of private health insurance, low maternal education and having carers who were not in the paid workforce in the last 12 months. In relation to perinatal factors, maternal age ≤ 19 years at childbirth and smoking in pregnancy were also associated with increased risk. Living in disadvantaged compared to more advantaged areas, and in more remote areas compared to more urban areas, was also associated with an increased risk of emotional maturity developmental vulnerability.

#### Non-aboriginal children

Non-Aboriginal boys were more than three times as likely as non-Aboriginal girls to be assessed as vulnerable (RR: 3.48, 95% CI 3.28–3.70). Other variables associated with increased risk of emotional developmental vulnerability amongst non-Aboriginal children included having a single parent, maternal age ≤ 19 years at childbirth (or 35 years and older at childbirth (compared to 30–34 years), low maternal education and having carers who were not in the paid workforce in the last 12 months. Maternal ages of 30–34 years at childbirth, parity of one and attending preschool in the year before school were associated with a decreased risk of emotional vulnerability amongst non-Aboriginal children.

### Inequalities in social and emotional development

Aboriginal children were twice as likely as non-Aboriginal children to be assessed as vulnerable socially (RR: 2.00, 95% CI: 1.89–2.12) and 89% more likely to be assessed as vulnerable emotionally (RR, 1.89, 95% CI: 1.77–2.02) (Table 2). In absolute terms, the sex- and year-adjusted risk differences were 7.7 percentage points (95% CI: 6.9–8.6) on the social competence domain and 4.9 percentage points (95% CI: 4.2–5.5) on the emotional maturity domain. After adjusting for differences in measures of socioeconomic advantage and disadvantage (Model 2), the relative risk was attenuated to 1.31 (95% CI: 1.23–1.40) on the social competence domain and 1.24 (95% CI: 1.15–1.33) on the emotional maturity domain (Table 2). After further adjusting for differences in perinatal factors (Model 3), the relative risks were further attenuated to 1.21 (95% CI: 1.14–1.29) for social competence and 1.16 (95% CI: 1.08–1.25) for emotional maturity (Table 2).

**Table 2:** Relative risks for social and emotional developmental vulnerability between Aboriginal and non-Aboriginal children in NSW

Model	Covariates adjusted for in each model:	Social competence	Emotional maturity
		RR (95% CI)	RR (95% CI)
1	Sex and AEDC census year.	2.00 (1.89, 2.12)	1.89 (1.77, 2.02)
2	M1 + health insurance/patient, mother partnered/single parent, maternal school education, highest occupation level of either parent, area-level socioeconomic disadvantage, and geographic remoteness	1.31 (1.23, 1.40)	1.24 (1.15, 1.33)
3	M2 + maternal age at childbirth, parity, smoking during pregnancy, antenatal care in the first 20 weeks, maternal comorbidity during pregnancy, and gestational age.	1.21 (1.14, 1.29)	1.16 (1.08, 1.25)

*RR* relative risk, *CI* confidence interval

#### Sensitivity analyses

Two sensitivity analyses were performed. To assess the impact of missing data we compared the study population to (i) the sample of children with complete covariates and (ii) the imputed sample (Table S2). This analysis showed that, compared to the complete-covariate sample, the imputed sample had a similar proportion of children with developmental vulnerability on the social competence and emotional maturity domains at age five while including the maximum available information in the analysis. To assess the impact of restricting to Public school children, we compared the restricted sample to all school children. This analysis confirmed that the restricted population of Public School children had a similar prevalence of developmental vulnerability among Aboriginal and non-Aboriginal children compared to the whole population.

## Discussion

In this population-based cohort study of more than 100,000 Australian children, we found that 84–88% of Aboriginal children were developing favourably, scoring above the developmental vulnerability threshold for social and emotional development on the AEDC measure. However, Aboriginal children were twice as likely to be vulnerable on measures of social and emotional development than their non-Aboriginal classmates. To our knowledge, this is the first study to identify child, family and area characteristics associated with social and emotional developmental vulnerability amongst Australian Aboriginal children at school entry. Importantly, a large proportion of the inequality in social and emotional development between Aboriginal and non-Aboriginal children was explained by differences in observed socioeconomic disadvantage and perinatal characteristics.

The child, family and area characteristics associated with a higher risk of vulnerability in relation to social competence and emotional maturity were largely similar amongst Aboriginal and non-Aboriginal children in our study population. Apart from the commonly noted elevated risk for social and emotional vulnerability among boys [2, 10, 29, 30], family-based measures of socioeconomic advantage and disadvantage, such as maternal education and parental occupation, were also associated with social and emotional development in Aboriginal and non-Aboriginal children. While there is no comparable data on Aboriginal social and emotional development from other studies, these findings accord with a small number of cohort studies examining social and emotional wellbeing amongst Aboriginal children of a similar age [2, 29], and with a large body of evidence describing the social determinants of physical and mental health and development across a range of populations [9–11, 31, 32].

For both Aboriginal and non-Aboriginal children, perinatal characteristics associated with an increased risk of social and emotional developmental vulnerability included: younger motherhood, higher parity, smoking in pregnancy, no antenatal care in the first 20 weeks of pregnancy, and younger gestational age at birth. While not previously documented amongst Aboriginal children, the association of this set of perinatal factors with social and emotional development is broadly consistent with those noted in other populations [32–34]. A more detailed exploration of the relationship between the whole distribution of gestational and maternal ages at birth, and physical, social, emotional, language, cognitive and communication development, has previously been documented in this study population [28, 33]. Our finding that Aboriginal children who lived in more disadvantaged or more geographically remote areas were at greater risk of being assessed as vulnerable in terms of social competence and emotional maturity also aligns with research in other populations [7]. Area-based characteristics such as social cohesion and access to resources such as parks, preschools and health and social services [31] have been hypothesised to impact on early childhood development, however, the mechanisms underpinning their impact have not been established [34].

An important finding of this study is that most, if not all, of the inequalities in social and emotional developmental vulnerability between Aboriginal and non-Aboriginal school entrants was attributable to the disproportionate burden of socioeconomic disadvantage that Aboriginal children experience from early life. This accords with a growing body of evidence documenting the major role that socioeconomic disadvantage plays in a range of outcomes for Aboriginal people [35, 36]. The multigenerational disadvantage many Aboriginal Australians experience is an ongoing legacy of colonisation and is known to underpin much of the mental and physical health disparity experienced by Aboriginal people of all ages [37]. The central role that disadvantage plays in vulnerable social and emotional development amongst Aboriginal children in early childhood underscores the need for targeted, culturally safe programs and support for disadvantaged children and families, to complement universal prevention efforts.

A key strength of the current study is the high population coverage of the source data and the large sample size afforded by examining two cohorts of school entrants in New South Wales, which made visible the social and emotional development experience of more than 7,000 Aboriginal children in early childhood. The use of linked, cross-sectoral population level data sources allowed us to examine, for the first time at a population-level, a wide range of early life characteristics of the child, their family and the area where they live that are related to social and emotional developmental vulnerability in Aboriginal children, and the factors that underlie inequalities in social and emotional development between Aboriginal and non-Aboriginal children. The use of multiple linked datasets, as in the current study, has been shown to increase enumeration of Aboriginal people [38]. Further, recall errors are minimised due to teacher (as opposed to parent) rating of development and midwives’ recording of data. On the other hand, teacher assessment, particularly cross-culturally, of the social competence and emotional maturity of Aboriginal children may introduce bias. Other studies using the AEDC, however, have demonstrated that the scores for Aboriginal children do not seem appreciably different when a cultural consultant is on hand to assist with the assessment [10, 20] and the AEDC has been validated within Australia and internationally, including an extensive evaluation for Australian Aboriginal children [20].

Three key limitations in the source data should be noted. First, the main result presented here pertains to the 65% of school entrants in New South Wales in the relevant years who attended public schools only, as school enrolment data (including parental employment and education information) was not available for non-Public School students. However, our sensitivity analysis showed a similar proportion of children were socially and emotionally vulnerable in our study population compared with all NSW Kindergarten children with available outcome data. Second, while the measures of socioeconomic advantage and disadvantage used in this study provided powerful insights into the factors underpinning emotional and social development amongst Aboriginal children, they were limited to those available in routinely collected data. Thus, we were unable to capture important aspects of intergenerational disadvantage such as forced removal of children and racism and discrimination [39, 40] or other important influences on child social and emotional development, such as the child’s physical health and parental mental and physical health and parenting style. A further minor limitation relates to the dichotomisation of the social competence and emotional maturity outcomes. Although it is standard practice to dichotomise the AEDC development outcomes for population-level research because of the skewed distribution of the raw scores, and this is standard practice in national reports [8] this results in a loss of information and over-simplifies the experience of social and emotional challenges to a binary presence or absence of vulnerability.

The results of the current study suggest that service providers might usefully seek to engage with Aboriginal communities around these data and work in partnership with them to develop culturally appropriate strategies for supporting Aboriginal families. Children from families which are identified in the antenatal period as experiencing factors associated with early childhood developmental vulnerability (such as parents not being in the paid workforce, preterm birth and living in a disadvantaged area) may be particularly likely to benefit from culturally appropriate support commensurate to need throughout early childhood. Our findings also highlight a vulnerability gap between children living in more and less advantaged or remote areas. Increasing access to services, recreational facilities and support in these areas have been suggested as potentially promising avenues for reducing developmental inequalities [41, 42]. It appears that the types of services required to improve the social and emotional development of Aboriginal children are not just those which focus directly on the child or on parenting but may also include those that improve the social and economic conditions that children grow up with. For example, support for parents to access further education and employment support, and improving access to the social determinants of health for Aboriginal children and their families including housing [37]. The Australian government’s recognition of the central importance of the social determinants of health is acknowledged by the inclusion of targets such as halving the gap between Aboriginal and non-Aboriginal Australians completing secondary school by 2020 (this target is on track) and halving the gap in employment by 2018 in the Close the Gap campaign (this target is on track). Over and above the aforementioned factors, efforts to address issues such as racism and discrimination to improve equality of access to opportunities for Aboriginal children and families are also important.

The current study is the largest and most comprehensive of social and emotional development among Australian Aboriginal children to date. It finds that while the substantial majority of Aboriginal children have social and emotional development above the developmental vulnerability threshold on the AEDC, Aboriginal children were twice as likely to be assessed as vulnerable. It demonstrates that almost all of the gap in early childhood social and emotional development between Aboriginal and non-Aboriginal children can be attributed to the disproportionate burden of socioeconomic and early life health disadvantage Aboriginal children and families experience. In addition, it provides important insights for policy and practice regarding the child, family and area-level characteristics of children most likely to benefit from enhanced, culturally appropriate, support. More broadly, our findings support the need for health and social policies and programs that aim to reduce the socioeconomic and health inequalities experienced by Aboriginal Australians from early life. The AEDC provides a unique opportunity to monitor the impact of policies on closing the gap in early childhood development between Aboriginal and non-Aboriginal children at a population-level.

## Additional file

Additional file 1: **Table S1.** Comparison of characteristics of Aboriginal and non-Aboriginal children in their first year of school in New South Wales in 2009 and 2012 for the study population with missing data, children with complete covariate data, and the study population with imputed data. (DOCX 59 kb)

## Data Availability

The data linked for this study are available from the national and state government data custodians for each administrative dataset, following the relevant ethical approvals. Details of the source data and record linkage for this study are available in the Seeding Success study protocol (BMJ Open, 2015) and data resource profile (Int J Epidemiol, 2017). Interested researchers should contact the NSW Centre for Health Record Linkage (http://www.cherel.org.au/ cherel.mail@moh.health.nsw.gov.au) regarding access to the source data, data custodian approval, and record linkage.

## Abbreviations

AEDC: Australian Early Development Census
AEDI: Australian Early Development Index
ARIA+: Accessibility/Remoteness Index of Australia
NSW: New South Wales
