# Supplementary material for: Social and emotional developmental vulnerability at age five in Aboriginal and non-Aboriginal children in New South Wales: a population data linkage study
Source: Int J Equity Health. 2019 Jul 31;18:120. doi: 10.1186/s12939-019-1019-x (PMC6668060; doi:10.1186/s12939-019-1019-x)
Supplement: Supplementary file 1 — Table S1. Comparison of characteristics of Aboriginal and non-Aboriginal children in their first year of school in New South Wales in 2009 and 2012 for the study population with missing data, children with complete covariate data, and the study population with imputed data. (DOCX 59 kb) [file 12939_2019_1019_MOESM1_ESM.docx]

**Supplementary Table 1: Comparison of characteristics of Aboriginal and non-Aboriginal children in their first year of school in New South Wales in 2009 and 2012 for the study population with missing data, children with complete covariate data, and the study population with imputed data**

|  | **Study population** | | | | | | **Sample with complete covariates** | | | | **Imputed sample** | | | |
| --- | --- | --- | --- | --- | --- | --- | --- | --- | --- | --- | --- | --- | --- | --- |
|  | Aboriginal^a^ | | | Non-Aboriginal | | | Aboriginal^a^ | | Non-Aboriginal | | Aboriginal^a^ | | Non-Aboriginal | |
|  | n | % of N | % of non-missing | n | % | % of non-missing | n | % | n | % | n | % | n | % |
| Total (N) | 7384 | 100 | 100 | 95104 | 100 | 100 | 4661 | 100 | 76936 | 100 | 7384 | 100 | 95104 | 100 |
| **Outcomes** |  |  |  |  |  |  |  |  |  |  |  |  |  |  |
| Social competence |  |  |  |  |  |  |  |  |  |  |  |  |  |  |
| Developmentally vulnerable | 1193 | 16 | 16 | 7780 | 8 | 8 | 665 | 14 | 5667 | 7 | 1194 | 16 | 7789 | 8 |
| Not developmentally vulnerable | 6183 | 84 | 84 | 87218 | 92 | 92 | 3988 | 86 | 71178 | 93 | 6190 | 84 | 87315 | 92 |
| Missing | 8 | <1 | - | 106 | <1 | - | - | - | - | - | - | - | - | - |
| Emotional maturity |  |  |  |  |  |  |  |  |  |  |  |  |  |  |
| Developmentally vulnerable | 883 | 12 | 12 | 6124 | 6 | 6 | 471 | 10 | 4541 | 6 | 888 | 12 | 6188 | 7 |
| Not developmentally vulnerable | 6463 | 88 | 88 | 88442 | 93 | 94 | 4175 | 90 | 71996 | 94 | 6496 | 88 | 88916 | 93 |
| Missing | 38 | 1 | - | 538 | 1 | - | - | - | - | - | - | - | - | - |
|  |  |  |  |  |  |  |  |  |  |  |  |  |  |  |
| **Socio-demographic characteristics** |  |  |  |  |  |  |  |  |  |  |  |  |  |  |
| Sex |  |  |  |  |  |  |  |  |  |  |  |  |  |  |
| Female | 3760 | 51 | 51 | 46784 | 49 | 49 | 2347 | 50 | 37885 | 49 | 3760 | 51 | 46784 | 49 |
| Male | 3624 | 49 | 49 | 48320 | 51 | 51 | 2314 | 50 | 39051 | 51 | 3624 | 49 | 48320 | 51 |
| Mother married/partnered^b^ |  |  |  |  |  |  |  |  |  |  |  |  |  |  |
| Yes | 3267 | 44 | 48 | 77330 | 81 | 84 | 2479 | 53 | 65870 | 86 | 3505 | 47 | 79302 | 83 |
| No | 3528 | 48 | 52 | 15195 | 16 | 16 | 2182 | 47 | 11066 | 14 | 3879 | 53 | 15802 | 17 |
| Missing | 589 | 8 | - | 2579 | 3 | - | - | - | - | - | - | - | - | - |
|  |  |  |  |  |  |  |  |  |  |  |  |  |  |  |
|  |  |  |  |  |  |  |  |  |  |  |  |  |  |  |
|  | **Study population** | | | | | | **Sample with complete covariates** | | | | **Imputed sample** | | | |
|  | Aboriginal^a^ | | | Non-Aboriginal | | | Aboriginal^a^ | | Non-Aboriginal | | Aboriginal^a^ | | Non-Aboriginal | |
|  | n | % of N | % of non-missing | n | % | % of non-missing | n | % | n | % | n | % | n | % |
| Private patient/insurance^b^ |  |  |  |  |  |  |  |  |  |  |  |  |  |  |
| Yes | 506 | 7 | 7 | 31272 | 33 | 33 | 427 | 9 | 27276 | 35 | 511 | 7 | 31579 | 33 |
| No | 6798 | 92 | 93 | 62848 | 66 | 67 | 4234 | 91 | 49660 | 65 | 6873 | 93 | 63525 | 67 |
| Missing | 80 | 1 | - | 984 | 1 | - | - | - | - | - | - | - | - | - |
| Maternal school education^c^ |  |  |  |  |  |  |  |  |  |  |  |  |  |  |
| 12 years | 1852 | 25 | 31 | 57226 | 60 | 65 | 1561 | 33 | 50889 | 66 | 2140 | 29 | 61148 | 64 |
| 10-11 years | 3003 | 41 | 50 | 25923 | 27 | 30 | 2320 | 50 | 22342 | 29 | 3820 | 52 | 28691 | 30 |
| ≤9 years | 1099 | 15 | 18 | 4620 | 5 | 5 | 780 | 17 | 3705 | 5 | 1424 | 19 | 5266 | 6 |
| Missing | 1430 | 19 | - | 7335 | 8 | - | - | - | - | - | - | - | - | - |
|  |  |  |  |  |  |  |  |  |  |  |  |  |  |  |
| Highest level of parental occupation^d^ |  |  |  |  |  |  |  |  |  |  |  |  |  |  |
| Managers/professionals | 561 | 8 | 9 | 23711 | 25 | 27 | 480 | 10 | 21149 | 27 | 613 | 8 | 24809 | 26 |
| Business managers/associated professionals | 727 | 10 | 12 | 21963 | 23 | 25 | 619 | 13 | 19469 | 25 | 816 | 11 | 23133 | 24 |
| Trades/clerks/services | 1368 | 19 | 22 | 22500 | 24 | 25 | 1103 | 24 | 19569 | 25 | 1607 | 22 | 24060 | 25 |
| Drivers/hospitality/labourers | 1700 | 23 | 27 | 14357 | 15 | 16 | 1267 | 27 | 11608 | 15 | 2049 | 28 | 15591 | 16 |
| Not in paid work in last 12 months | 1840 | 25 | 30 | 6739 | 7 | 8 | 1192 | 26 | 5141 | 7 | 2299 | 31 | 7511 | 8 |
| Missing | 1188 | 16 | - | 5834 | 6 | - | - | - | - | - | - | - | - | - |
|  |  |  |  |  |  |  |  |  |  |  |  |  |  |  |
| Area-level disadvantage^e^ |  |  |  |  |  |  |  |  |  |  |  |  |  |  |
| Quintile 5 (Least disadvantaged) | 372 | 5 | 5 | 23613 | 25 | 25 | 270 | 6 | 20207 | 26 | 378 | 5 | 23884 | 25 |
| Quintile 4 | 904 | 12 | 12 | 19980 | 21 | 21 | 639 | 14 | 16634 | 22 | 914 | 12 | 20211 | 21 |
| Quintile 3 | 3043 | 41 | 42 | 32314 | 34 | 34 | 1990 | 43 | 25860 | 34 | 3096 | 42 | 32697 | 34 |
| Quintile 2 | 1367 | 19 | 19 | 9951 | 10 | 11 | 841 | 18 | 8040 | 10 | 1389 | 19 | 10072 | 11 |
| Quintile 1 (Most disadvantaged) | 1582 | 21 | 22 | 8138 | 9 | 9 | 921 | 20 | 6195 | 8 | 1607 | 22 | 8240 | 9 |
| Missing | 116 | 2 | - | 1108 | 1 | - | - | - | - | - | - | - | - | - |
|  |  |  |  |  |  |  |  |  |  |  |  |  |  |  |
|  | **Study population** | | | | | | **Sample with complete covariates** | | | | **Imputed sample** | | | |
|  | Aboriginal^a^ | | | Non-Aboriginal | | | Aboriginal^a^ | | Non-Aboriginal | | Aboriginal^a^ | | Non-Aboriginal | |
|  | n | % of N | % of non-missing | n | % | % of non-missing | n | % | n | % | n | % | n | % |
| Geographic remoteness^f^ |  |  |  |  |  |  |  |  |  |  |  |  |  |  |
| Major City | 2772 | 38 | 38 | 62133 | 65 | 66 | 1890 | 41 | 50659 | 66 | 2815 | 38 | 62858 | 66 |
| Inner Regional | 2535 | 34 | 35 | 23702 | 25 | 25 | 1598 | 34 | 19661 | 26 | 2578 | 35 | 23985 | 25 |
| Outer Regional | 1531 | 21 | 21 | 7616 | 8 | 8 | 953 | 20 | 6168 | 8 | 1554 | 21 | 7709 | 8 |
| Remote/Very Remote | 430 | 6 | 6 | 545 | 1 | 1 | 220 | 5 | 448 | 1 | 437 | 6 | 552 | 1 |
| Missing | 116 | 2 | - | 1108 | 1 | - | - | - | - | - | - | - | - | - |
| Attended preschool/childcare |  |  |  |  |  |  |  |  |  |  |  |  |  |  |
| Yes | 5438 | 74 | 81 | 79480 | 84 | 89 | 3870 | 83 | 69072 | 90 | 5974 | 81 | 84547 | 89 |
| No | 1267 | 17 | 19 | 9671 | 10 | 11 | 791 | 17 | 7864 | 10 | 1410 | 19 | 10557 | 11 |
| Missing | 679 | 9 | - | 5953 | 6 | - | - | - | - | - | - | - | - | - |
|  |  |  |  |  |  |  |  |  |  |  |  |  |  |  |
| AEDC census year |  |  |  |  |  |  |  |  |  |  |  |  |  |  |
| 2009 | 3325 | 45 | 45 | 45180 | 48 | 48 | 2137 | 46 | 37061 | 48 | 3325 | 45 | 45180 | 48 |
| 2012 | 4059 | 55 | 55 | 49924 | 52 | 52 | 2524 | 54 | 39875 | 52 | 4059 | 55 | 49924 | 52 |
|  |  |  |  |  |  |  |  |  |  |  |  |  |  |  |
| ***Perinatal characteristics*** |  |  |  |  |  |  |  |  |  |  |  |  |  |  |
| Maternal age at childbirth |  |  |  |  |  |  |  |  |  |  |  |  |  |  |
| <20 | n.p. | 17 | 17 | n.p. | 3 | 3 | 674 | 14 | 2093 | 3 | 1254 | 17 | 3190 | 3 |
| 20-24 | 2254 | 31 | 31 | 13870 | 15 | 15 | 1383 | 30 | 10423 | 14 | 2254 | 31 | 13871 | 15 |
| 25-29 | 1856 | 25 | 25 | 26176 | 28 | 28 | 1225 | 26 | 21388 | 28 | 1856 | 25 | 26177 | 28 |
| 30-34 | 1344 | 18 | 18 | 31697 | 33 | 33 | 912 | 20 | 26493 | 34 | 1344 | 18 | 31698 | 33 |
| ≥35 | 675 | 9 | 9 | 20167 | 21 | 21 | 467 | 10 | 16539 | 21 | 675 | 9 | 20167 | 21 |
| Missing | n.p. | <1 | - | n.p. | <1 | - | - | - | - | - | - | - | - | - |
|  |  |  |  |  |  |  |  |  |  |  |  |  |  |  |
|  |  |  |  |  |  |  |  |  |  |  |  |  |  |  |
|  |  |  |  |  |  |  |  |  |  |  |  |  |  |  |
|  | **Study population** | | | | | | **Sample with complete covariates** | | | | **Imputed sample** | | | |
|  | Aboriginal^a^ | | | Non-Aboriginal | | | Aboriginal^a^ | | Non-Aboriginal | | Aboriginal^a^ | | Non-Aboriginal | |
|  | n | % of N | % of non-missing | n | % | % of non-missing | n | % | n | % | n | % | n | % |
| Parity |  |  |  |  |  |  |  |  |  |  |  |  |  |  |
| 0 | 2437 | 33 | 34 | 38880 | 41 | 41 | 1662 | 36 | 31894 | 41 | 2475 | 34 | 39395 | 41 |
| 1 | 2018 | 27 | 28 | 32641 | 34 | 35 | 1354 | 29 | 27427 | 36 | 2057 | 28 | 33081 | 35 |
| ≥2 | 2799 | 38 | 39 | 22311 | 23 | 24 | 1645 | 35 | 17615 | 23 | 2852 | 39 | 22628 | 24 |
| Missing | 130 | 2 | - | 1272 | 1 | - | - | - | - | - | - | - | - | - |
| Smoking during pregnancy |  |  |  |  |  |  |  |  |  |  |  |  |  |  |
| No | 3761 | 51 | 52 | 80297 | 84 | 86 | 2737 | 59 | 67561 | 88 | 3835 | 52 | 81685 | 86 |
| Yes | 3484 | 47 | 48 | 13217 | 14 | 14 | 1924 | 41 | 9375 | 12 | 3549 | 48 | 13419 | 14 |
| Missing | 139 | 2 | - | 1590 | 2 | - | - | - | - | - | - | - | - | - |
| Antenatal care in first 20 weeks |  |  |  |  |  |  |  |  |  |  |  |  |  |  |
| No | 1356 | 18 | 19 | 9916 | 10 | 11 | 744 | 16 | 7408 | 10 | 1430 | 19 | 10153 | 11 |
| Yes | 5706 | 77 | 81 | 83226 | 88 | 89 | 3917 | 84 | 69528 | 90 | 5954 | 81 | 84951 | 89 |
| Missing | 322 | 4 | - | 1962 | 2 | - | - | - | - | - | - | - | - | - |
| Maternal comorbidity^g^ |  |  |  |  |  |  |  |  |  |  |  |  |  |  |
| No | 6592 | 89 | 90 | 83916 | 88 | 89 | 4174 | 90 | 68420 | 89 | 6682 | 90 | 84721 | 89 |
| Yes | 693 | 9 | 10 | 10280 | 11 | 11 | 487 | 10 | 8516 | 11 | 702 | 10 | 10383 | 11 |
| Missing | 99 | 1 | - | 908 | 1 | - | - | - | - | - | - | - | - | - |
| Gestational age group |  |  |  |  |  |  |  |  |  |  |  |  |  |  |
| Early preterm (22-33 weeks) | 185 | 3 | 3 | 1383 | 1 | 1 | 107 | 2 | 995 | 1 | 187 | 3 | 1397 | 1 |
| Late preterm (34-36 weeks) | 528 | 7 | 7 | 4417 | 5 | 5 | 293 | 6 | 3478 | 5 | 533 | 7 | 4464 | 5 |
| Early term (37-38 weeks) | 1646 | 22 | 23 | 20786 | 22 | 22 | 1014 | 22 | 16999 | 22 | 1669 | 23 | 20995 | 22 |
| Full to postterm (≥39 weeks) | 4924 | 67 | 68 | 67592 | 71 | 72 | 3247 | 70 | 55464 | 72 | 4995 | 68 | 68248 | 72 |
| Missing | 101 | 1 | - | 926 | 1 | - | - | - | - | - | - | - | - | - |
| There were no missing values for sex or AEDC census year. The counts for the imputed data are an average across the 5 complete datasets. ^a^ Defined as child or parent identified as Aboriginal on any of the birth records (i.e. perinatal data collection, birth registration or hospital birth record), or AEDC school record; ^b^ Based on hospital birth record; ^c^ Highest level of education of mother or carer recorded on school enrolment; ^d^ Based on highest ranking occupation of either parent or carer recorded on school enrolment; ^e^ Socio-Economic Indices for Areas (SEIFA) Index of Relative Socio-economic Advantage and Disadvantage population quintiles based on mother’s statistical local area of residence at the time of birth; ^f^ Accessibility/Remoteness Index of Australia (ARIA+) based on mother’s statistical local area of residence at the time of birth; ^g^ Includes pre-existing and gestational-onset diabetes and hypertension. | | | | | | | | | | | | | | |
